# Draft Genome Sequence of Campylobacter fetus subsp. *fetus* CITCf01, Isolated from a Patient with Subacute Bacterial Endocarditis

**DOI:** 10.1128/MRA.01556-18

**Published:** 2019-03-14

**Authors:** Eamonn P. Culligan, James O’Connor, Caoimhe Lynch, Deirdre O’Brien, Carl Vaughan, Declan Bolton, Aidan Coffey, Roy D. Sleator, Brigid Lucey

**Affiliations:** a Department of Biological Sciences, Cork Institute of Technology, Bishopstown, Cork, Ireland; b Department of Clinical Microbiology, Mercy University Hospital, Grenville Place, Cork, Ireland; c Department of Cardiology, Mercy University Hospital, Grenville Place, Cork, Ireland; d Teagasc Food Research Centre, Ashtown, Dublin, Ireland; Indiana University, Bloomington

## Abstract

Campylobacter fetus is a Gram-negative, zoonotic pathogen and a member of the class *Epsilonproteobacteria*. We report the draft genome sequence of C. fetus subsp. fetus CITCf01, isolated from a patient with subacute bacterial endocarditis. CITCf01 grew under aerobic, microaerobic, and anaerobic conditions, and at 42°C, an unusual combination of growth conditions.

## Abstract (precis)

Campylobacter fetus is a Gram-negative, zoonotic pathogen and a member of the class *Epsilonproteobacteria*. We report the draft genome sequence of C. fetus subsp. fetus CITCf01, isolated from a patient with subacute bacterial endocarditis.

## ANNOUNCEMENT

Campylobacter fetus is a pathogen of animals and humans. Three subspecies have been characterized to date, namely C. fetus subsp. fetus, C. fetus subsp. venerealis, and C. fetus subsp. testudinum. C. fetus subsp. testudinum is primarily associated with reptiles (1, 2), while C. fetus subsp. fetus and C. fetus subsp. venerealis are associated with human and animal infections (3, 4). Invasive C. fetus infections in humans have included meningitis, prosthetic hip joint infection, bacteremia, pericarditis, endocarditis, and infectious aortitis, among others (5–11).

C. fetus subsp. fetus CITCf01 (hereafter referred to as CITCf01) was isolated from three peripheral blood culture sets (FAN BacT/Alert, bioMérieux) taken 1 day apart, after informed patient consent was obtained. The Virtuo BacT/Alert (bioMérieux) blood culture microbial detection system flagged both aerobic and anaerobic bottles positive for each set. The time-to-positivity (TTP) ranged from 40 hours 43 min to 62 hours 24 min (mean TTP 52 hours 8 min). Gram stain of cultures from each bottle were reported as curved, Gram-negative bacilli, typical of *Campylobacter* species.

Subcultures of the positive culture bottles grew microaerobically (at 35°C, 37°C, and 42°C) on selective charcoal-cefoperazone deoxycholate agar (CCDA) (Oxoid). CITCf01 grew in a 5% CO_2_-enriched aerobic environment, microaerobically and anaerobically, at 35°C on Columbia blood agar (CBA) (Oxoid) and on Mueller-Hinton agar. Furthermore, CITCf01 was cultured microaerobically from frozen stocks on blood-free *Campylobacter* selectivity agar (Sigma-Aldrich, USA). Subculturing on CBA confirmed weak growth under aerobic conditions at 37°C. The initial clinical identification of CITCf01 was by Vitek 2 biochemical analytical profile using the *Neisseria*-*Haemophilus* (NH) identification card and by matrix-assisted laser desorption ionization–time of flight (MALDI-TOF) mass spectrometry (Bruker Daltronics).

CITCf01 genomic DNA was isolated using the GenElute genomic DNA kit (Sigma-Aldrich, USA), in accordance with the manufacturer’s instructions. Library preparation, whole-genome sequencing (WGS), and subsequent assembly were performed by MicrobesNG (University of Birmingham, UK) using the following methods.

DNA was quantified in triplicate with the Quant-iT double-stranded DNA (dsDNA) high sensitivity assay kit (Thermo Fisher, USA). Genomic DNA libraries were prepared using the Nextera-XT protocol (Illumina, San Diego, USA) with the following exceptions: 2 ng of input DNA was used instead of 1 ng, and PCR elongation time was increased from 30 seconds to 1 min. DNA quantification and library preparation were performed on a Hamilton Microlab STAR system. Pooled libraries were quantified using the Kapa Biosystems library quantification kit on a Roche light cycler 96 quantitative PCR (qPCR) machine. Libraries were sequenced on the Illumina HiSeq using a 250-bp paired-end protocol. Reads were adapter trimmed using Trimmomatic 0.30 with a sliding window quality cutoff of Q15 (12). *De novo* assembly was performed on samples using SPAdes version 3.7.0 (13). Quality of assembly and predicted genome size were assessed using QUAST (14). WGS generated 75,582 reads, of which 72,785 passed quality filtering. Genome assembly generated a total of 55 contigs with a mean coverage of 18.96× and 42 contigs >200 bp. Following removal of low-coverage (<1.0×) contigs, the predicted genome size is approximately 1.75 Mb, with a G+C content of 33.22%. The genome was annotated by using the NCBI Prokaryotic Genome Annotation Pipeline (PGAP) (15) and is predicted to contain 1,778 genes (1,707 coding genes).

### Data availability.

This whole-genome shotgun project has been deposited at DDBJ/ENA/GenBank under the accession number RBHV00000000. The version described in this paper is the first version, RBHV01000000. Raw reads were submitted to SRA and are available via accession number PRJNA493797.
